# Motivational Factors Underlying Problem Solving: Comparing Wolf and Dog Puppies' Explorative and Neophobic Behaviors at 5, 6, and 8 Weeks of Age

**DOI:** 10.3389/fpsyg.2017.00180

**Published:** 2017-02-09

**Authors:** Sarah Marshall-Pescini, Zsófia Virányi, Enikő Kubinyi, Friederike Range

**Affiliations:** ^1^Comparative Cognition, Messerli Research Institute, University of Veterinary Medicine, Vienna, Medical University of Vienna, University of ViennaVienna, Austria; ^2^Wolf Science CentreErnstbrunn, Austria; ^3^Department of Ethology, Eötvös Loránd UniversityBudapest, Hungary; ^4^MTA-ELTE Comparative Ethology Research GroupBudapest, Hungary

**Keywords:** domestication, problem-solving, wolves, dogs, exploration, neophobia, critical period, development

## Abstract

**Background:** Wolves have been shown to be better in independent problem-solving tasks than dogs, however it is unclear whether cognitive or motivational factors underlie such differences. In a number of species problem solving has been linked to both persistence in exploration and neophobia, suggesting both these aspects may underlie dog-wolf differences in problem solving. Indeed adult wolves have been shown to be more likely to approach a novel object and more persistent in their investigation of it, but also slower in making contact with it and more fearful of it than dogs.

**Methods:** In the current study we investigated potential differences in equally-raised dogs' and wolves' explorative and neophobic behaviors in a novel environment and with novel objects at 5, 6, and 8 weeks of age.

**Results:** Results showed that wolves were more persistent in exploring both the environment and the objects than dogs, and this was the case at all ages. There were no differences in the frequency of fear-related behaviors and time spent in proximity to humans. Stress-related behaviors were similarly expressed at 5 and 6 weeks, although wolves showed a higher frequency of such behaviors at 8 weeks.

**Discussion:** Overall, results with puppies confirm those with adult animals: wolves appear to be more explorative than dogs. Such motivational differences need to be taken into account when comparing dogs and wolves in cognitive tasks.

## Introduction

Innovative problem solving refers to the acquisition of a novel behavior, which may allow an individual to exploit a new resource or environment (Ramsey et al., 2007; see also Reader and Laland, 2003). Variation in innovative problem solving both within and across species has been linked to cognitive abilities (Reader, 2003; Lefebvre et al., 2004; Griffin et al., 2014). However, motivational factors, in particular *persistence in exploration* and *neophobia*, are also related to success in problem solving tasks (Reader, 2003; Reader and Laland, 2003; Stöwe et al., 2006a,b). Explorative behavior, measured mostly in terms of the time spent (persistence in) investigating and interacting with an object/task, has been consistently shown to relate to problem solving success across a wide range of species (great tits: Morand-Ferron et al., 2011; blue tits: Morand-Ferron and Quinn, 2011; hyenas: Benson-Amram and Holekamp, 2012; meerkats: Thornton and Samson, 2012). Furthermore, in a number of bird species it was found that individuals who were least reluctant to approach novel objects (i.e., showed less neophobia) were also the quickest to solve novel foraging tasks (Webster and Lefebvre, 2001; Seferta et al., 2001; Auersperg et al., 2011). Similarly, in wild hyenas less neophobic individuals were significantly more successful than more neophobic ones in accessing a food puzzle box (Cole et al., 2011 for no links between neophobia and problem solving success; Benson-Amram and Holekamp, 2012, but see also Biondi et al., 2010).

A number of hypotheses posit that domestication may have negatively affected dogs' independent problem solving abilities (Frank, 1980; Frank and Frank, 1982, 1985; Frank et al., 1989; Frank, 2011) supported by studies showing that wolves are more successful than dogs in different tasks both as puppies (Frank and Frank, 1982, 1985; Frank et al., 1989) and adults (Hiestand, 2011). These differences in problem-solving skills have been related to differences in wolves' and dogs' cognitive complexity (e.g., Frank and Frank, 1985) and to dogs' greater sensitivity to social inhibition when tested with humans present (Topál et al., 1997; Udell, 2015).

Indeed when humans are present during problem solving tasks, dogs consistently behave differently compared to wolves. In the first studies comparing equally raised wolves and dogs, Frank and Frank (1985) presented pups with puzzle boxes of increasing complexity, noting that wolf pups were overall significantly more successful in obtaining the reward. Interestingly however, authors describe wolves “attacking each puzzle immediately” (pp. 271) and persisting “until the problem was solved or time ran out” (pp. 271) in contrast to dogs quickly reverting to seeking human attention upon discovering that the food was not immediately available, and then laying down until time elapsed. Similarly, when testing 4-month-old wolves and pet dogs in a manipulation task that suddenly became unsolvable, dogs quickly looked back toward the human handler whereas wolves did not (Miklósi et al., 2003). More recently, Udell (2015) presented adult wolves and dogs with a problem solving task and found that wolves were more persistent (and consequently more successful) than both pet and shelter dogs, regardless of human encouragement received during testing, whereas dogs consistently looked longer toward the human. Hence, taken together results suggest that when a human is present in the room, dogs show less persistent behavior than wolves in object manipulation tasks.

Interestingly however, differences between wolves and dogs in such tasks also emerge when humans are not present during testing. Udell (2015) tested adult dogs and wolves also when alone, and found wolves to be more persistent (and successful) in their efforts to obtain the reward. Furthermore, in a study comparing adult and 6-month-old wolves' and dogs' performance in a string-pulling task, differently from dogs, wolves required no prior training to solve the task, showed a greater variety of behaviors exhibited on the rope and in general were more persistent (Hiestand, 2011).

Hence, it appears that motivational factors and the explorative tendencies of wolves and dogs *per-se* (independently from human presence) differ, which may play a major role in the observed differences in problem solving abilities, as was indeed hinted at already in Frank and Frank's writing (“higher curiosity and explorative drive” (pp. 272, Frank and Frank, 1985) in wolves than in dogs).

Despite the potential significance explorative behaviors have for problem solving, few studies have directly investigated these tendencies in wolves and dogs. Early studies comparing wolves and dogs led most authors to agree that despite socialization, wolves are more neophobic than dogs (Fentress, 1967; Klinghammer and Goodmann, 1987; Zimen, 1987), but these descriptions contrast with those of wolf pups immediately “attacking” new puzzle boxes in experimental settings (Frank and Frank, 1985). This contradiction might be explained by focusing on different aspects of whether and how animals approach novelty. A recent study testing human-raised pack-living adult wolves and dogs kept in a game park setting under identical conditions, compared their reactions to novel objects and found that dogs were less likely to approach a novel object than wolves (i.e., only 7 of the 13 dogs but all 11 wolves approached the object), and wolves were more persistent in their investigation of the objects than dogs. At the same time however, wolves were slower in making contact with the object and fled from it more often than dogs (Moretti et al., 2015). Hence, it appears that adult wolves approach novel objects in their environment slower than dogs and they do so in a more cautious and fearful manner, but they then spend more time investigating it. Unequivocally however, wolves appear more interested in novel objects in their environment than dogs. Confirming this difference, another study found that wolves, at the age of 4 months, spent more time exploring an unfamiliar outdoor kennel than dogs (Topál et al., 2005). As outlined above, such an elevated motivation to approach and explore novel objects and places is likely to increase success also in problem solving. Interestingly, however Gácsi et al. (2005) found no differences in wolves and dogs' approach behavior to novel objects at 3 and 4 weeks: the animals moved in an unfamiliar room and approached different objects and persons in a similar way. This may suggest that at early ages dogs and wolves explore their environment similarly and only later (possibly between the ages of 1 and 4 months) dogs lose their interest in novel objects as compared to wolves.

Hence, to further elucidate the potential effects of domestication on dogs' explorative and neophobic behaviors at an early age, in the current study, we presented identically-raised and kept wolf and dog puppies at 5, 6, and 8 weeks of age, with a novel environment and a novel object test. In order to address neophobia in different ways, we recorded not only (1) likelihood and (2) latency to contact the novel objects but also (3) time spent exhibiting fear-related behaviors such as freezing and running around with a lowered posture (i.e., “escape” see **Table 2**); (4) the frequency of behaviors commonly associated with stress in these species (e.g., panting, yawning, scratching etc.) and the (5) time spent in proximity to the exit. Explorative behaviors we measured in terms of the (1) time the animals spent moving around in a novel environment maintaining a relaxed body posture and (2) actively investigating and playing with a novel object.

Based on results of the adult wolf-dog comparison, we hypothesized that already at this young age, wolves would show greater interest than dogs in exploring a new environment and a novel object, but that they would also show higher levels of stress and fear-related behaviors.

## Materials and methods

### Ethical statement

No special permission for use of animals (wolves and dogs) in such socio-cognitive studies is required in Austria (Tierversuchsgesetz 2012–TVG 2012). The relevant committee that allows running research without special permissions regarding animals is: Tierversuchskommission am Bundesministerium für Wissenschaft und Forschung (Austria).

### Subjects

Overall 17 wolves (6 F, 11 M) and 18 mixed-breed dogs (9 F, 9 M) housed at the Wolf Science Center (WSC, www.wolfscience.at) participated in the tests. All dogs and all wolves were tested in the novel object test, but only 14 dogs and 10 wolves were tested in the novel environment (Table 1). This was due to a number of reasons amongst which the sickness of a number of animals during the required testing period and in some cases the unavailability of a novel environment in which to conduct the test. A number of sibling pairs were tested, which is indicated in Table 1.

**Table 1:** **Tests carried out by each subject**.

**Name**	**Species**	**Sex**	**Cohort**	**Novel environment test**	**Novel object test**
Alika^1^	Dog	F	1	Y	Y
Asali^2^	Dog	M	2	Y	Y
Bashira^3^	Dog	F	2	Y	Y
Binti^2^	Dog	F	2	Y	Y
Bora^4^	Dog	F	3	N	Y
Doa^5^	Dog	F	1	Y	Y
Hakima^3^	Dog	M	2	Y	Y
Imani^6^	Dog	M	1	Y	Y
Jini^5^	Dog	F	1	Y	Y
Kali	Dog	M	3	N	Y
Kilio^7^	Dog	M	1	Y	Y
Layla^4^	Dog	F	3	N	Y
Maisha^7^	Dog	M	1	Y	Y
Meru^8^	Dog	M	2	Y	Y
Nia	Dog	F	3	N	Y
Rafiki^1^	Dog	M	1	Y	Y
Tana^8^	Dog	M	2	Y	Y
Uzima^6^	Dog	F	1	Y	Y
Amarok^9^	Wolf	M	4	N	Y
Apache^10^	Wolf	M	2	Y	Y
Aragorn^11^	Wolf	M	1	Y	Y
Cherokee^10^	Wolf	M	2	Y	Y
Chitto^12^	Wolf	M	4	N	Y
Geronimo^13^	Wolf	M	2	Y	Y
Kaspar	Wolf	M	1	Y	Y
Kay^14^	Wolf	F	4	N	Y
Kenai^15^	Wolf	M	3	Y	Y
Nanuk	Wolf	M	2	Y	Y
Shima^11^	Wolf	F	1	Y	Y
Tala^9^	Wolf	F	4	N	Y
Tatonga	Wolf	F	2	N	Y
Una^12^	Wolf	F	4	N	Y
Wamblee^14^	Wolf	M	4	N	Y
Wapi^15^	Wolf	M	3	Y	Y
Yukon^13^	Wolf	F	2	Y	Y

*Siblings are indicated by matching numerical superscript to their name. Animals raised in the same cohort are indicated by matching numbers*.

Dogs and wolves at the WSC are raised and kept in the same way, living in conspecific packs but with substantial interaction with human partners. At the time of testing, puppies lived in conspecific peer-groups, with 24 h a day contact to a human hand-raiser. Dog and wolf pups lived in a game park setting having access to a large outdoor enclosure and an indoor area where they slept together with the hand-raiser. Pups were regularly visited by unfamiliar people and were brought different toys and other objects they could interact with. Behavioral testing started at the age of 3 weeks, for which they were regularly moved to other rooms in separation from their peers (see Range and Virányi, 2011 for a full description of raising procedures).

### Overall procedure

The novel environment test was conducted at 5 weeks of age, whereas the novel object test was carried out twice, at 6 and at 8 weeks of age (see below). The two tests took place in two different rooms of the same size (3 m × 3 m). Both rooms were completely novel for the pups, but since the novel object test was conducted twice in the same room, it might have been at least somewhat familiar to the subjects after the first test. In both tests, the camera person was placed in such a way as to be out of sight/unavailable to the pups, either filming through a window or perched above a table/high bench. The camera person adopted her position prior to the pups' arrival and subsequently remained motionless and silent during the whole test.

### Novel environment test (at 5 weeks)

The hand-raiser brought the pup into the room holding it in her arms, then put it down in the center of the room and left. The pup remained in the room for 5 min.

### Novel object test (at 6 and 8 weeks)

**Figure 1:**
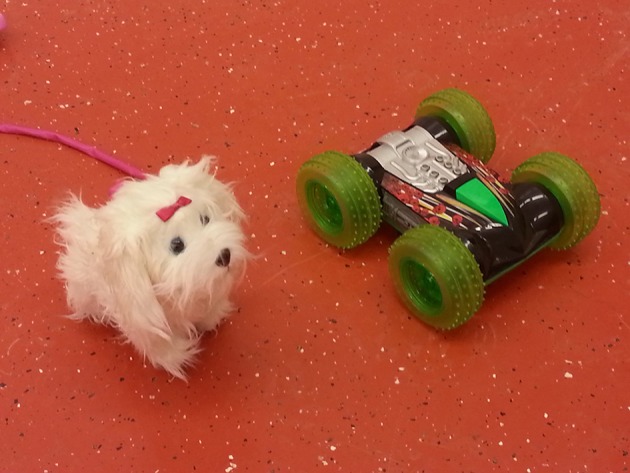
**Novel objects used in the test: the remote controlled car and toy dog**.

Two novel objects were used: (1) a toy dog which could be activated via a cord, and (2) a remote controlled car either with a cardboard box on top or not (see Figure 1).

The toy dog was presented to all puppies once at 6 weeks and once at 8 weeks, while the car was presented on one occasion without the box and on the other occasion with the box on top (henceforth “car-box”). For all subjects, the toy-dog was presented prior to the car/car-box, whereas the presentation order of the car vs. car-box was counterbalanced across subjects.

At 6 and 8 weeks the pups were exposed to a series of consecutive tests carried out on the same day including the novel objects test. All pups were exposed to the same tests in the same order, i.e., (1) pup alone in the room with a stranger, (2) recall test by the stranger, (3) novel object: toy-dog (4) fetch and retrieve with stranger (5) novel object: car/car-box. The procedures for the novel object tests were the same for both objects. The object was placed in the middle of the room whilst switched off. The experimenter (a female stranger) was standing against the wall holding in her hand either the cord to which the toy dog was attached, or the remote controller of the car. An empty chair was placed next to the wall opposite the experimenter. Then, the caregiver/trainer carried the pup into the room and placed it on the floor 1.5 m away from the novel object and then left the room. The test involved two phases (30 s each). Phase 1 started as soon as the pup was placed on the ground. At this point the experimenter activated either the toy-dog (which started intermittently barking and walking with jerky movements) or the car that the experimenter remotely moved away from the pup, then toward it and then parallel to it. The objects continued moving for a total of 30 s. In Phase 2 the toy dog/car stopped moving and remained stationary for 30 s.

### Coding and analyses

All videos were coded using the Solomon Coder (Version Solomon beta 100926, copyright András Péter). Behaviors coded are summarized in Table 2.

**Table 2:** **Behavioral categories and single behaviors, including definitions, coded in each test**.

	**Behavioral coding**		
	**Description**	**Test**	**Measure**
Exploration	To walk or run, including any activity (sniffing, distal and close visual inspection or oral examination) directed toward the environment in a relaxed manner. That is, tail is either wagging perpendicularly or held in a neutral position, ears are pointed forward, body posture is relaxed.	NE, NO	Dur
Self-play	To run around in the testing area, exhibiting exaggerated behaviours such as bounces and stop-starts and play bows. Often accompanied by tail wagging.	NE	Dur
Contact object	First contact (with nose, or paw) between the animal and the object. Latency measured from the start of the test (when the hand-raiser closed the door) to first contact.	NO	Lat
Object interaction	Moving the object or parts of it actively (e.g., pulling on the toy dog's tail, biting it without moving the whole dog) and/or playing with the object, i.e., run around it, snapping, jumping, pawing or barking at it (including play bowing), accompanied with erected ears and often tail wagging.	NO	Dur, Freq
Contact Experimenter	First contact during the entire experiment between pup and experimenter. Latency measured from the start of the test (hand-raiser closing the door) to first contact.	NO	Lat
**PROXIMITY PEOPLE**			
Close to cameraperson	The subject is in front of the cameraman (on occasions combined with climbing movements on the wall/table/bench directly in front/below of the camera person).	NE, NO	Dur
Close to experimenter	The time spent within 2 body lengths of the Experimenter.	NO	Dur
Stress-related behaviors	The sum of the following:		
Licking	Tongue moved over the lips.	NE, NO	Freq
Panting	To gasp for breath. The tongue is visibly moving inside and outside the mouth.	NE, NO	Freq
Scratching	To nibble (autogrooming) or scratch different body parts with front or hind paws.	NE, NO	Freq
Shaking	To wiggle the whole body, starting with the head and finishing with the hind part of the body.	NE, NO	Freq
Yawning	To open the mouth widely, slightly close the eyes and backward the ears. Sometimes accompanied by yawning noises.	NE, NO	Freq
Fear-related behaviors	The sum of the following:		
Escape	To walk or run with tail tucked and often body ducked down. A tense, crouched body posture is often combined with lowered head and ears held back. This category also includes climbing movements on the wall, door or exit construction.	NE, NO	Dur
Climb	Trying to climb on Experimenter.	NO	Dur
Freeze	The pup stops moving and is staring at the source of fear.	NO	Dur
Close to entry/exit	The subject is within 2 body lengths of the door (often combined with scratching movements-but this is not required).	NE, NO	Dur
Vocalizations	To whine, whimper or howl.	NE, NO	Freq

*NE, Novel environment test; NO, Novel object test. Measures taken for each behavior are specified Dur, Duration (s); Freq, Frequency; Lat, latency (s)*.

For the novel environment test general linear models (GLM) were conducted, either with the relative duration (over total test time), latency or frequencies of behaviors as response variables and species as the independent variable.

A GLMM using binomial distribution was run to assess the likelihood (i.e., the number of animals) of contacting the object in relation to age and species. Analyses on latency, frequency, and duration of interacting with the object were carried out only for those animals that exhibited the behavior, similarly latency to contact the experimenter was also analyzed only for animals that did in fact contact her. Generalized linear mixed models (GLMM) were run with the relative durations (over total test time, which varied somewhat across subjects), latency or frequencies of behaviors as response variables and species and age at testing as independent variables (including the interaction between them), and the subject as random factor. For all analyses we adopted a model reduction approach, dropping least significant items one at a time, starting from interaction effects. All analyses were run in R (version 3.2), frequencies, latencies, and likelihoods were run in the package lme4, whereas models with relative durations as the response variables were run in package nlme (for the latter models a quasi-binomial distribution was used, because the error distribution was between 0 and 1) (www.r-project.org).

## Results

### Novel environment test

**Figure 2:**
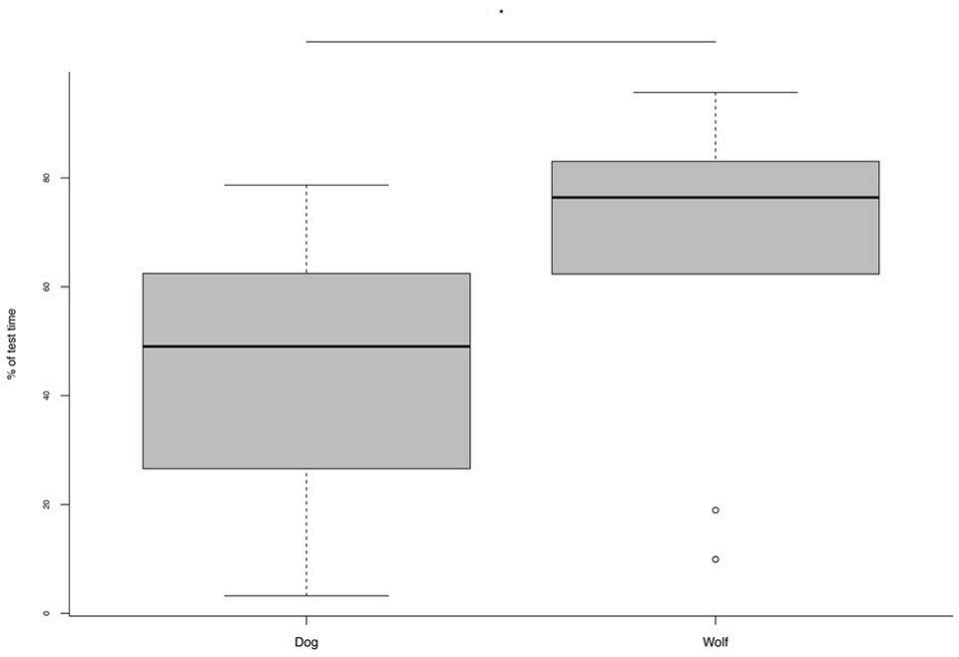
**Percentage of trial time spent exploring the new environment for dogs and wolves in the novel environment test**. ^*^p = 0.01.

In the novel environment test, the only significant difference emerging was that wolves spent more time carrying out explorative behaviors than dogs (see Table 3; Figure 2).

**Table 3:** **Results of the species comparison in the “Novel environment” test (no of wolves = 10; no of dogs = 14)**.

**Behaviour**	**Measure**	**Model (glmm) statistics**
**Exploration**	D	***F***_1_ = **7.116**, ***p*** = **0.014**
Self-play	L	*z* = 0.975, *p* = 0.330
Stress-related behaviours	F^∧^	*t* = 1.444, *p* = 0.163
Escape behaviours	D	*F*_1_ = 0.065, *p* = 0.801
Vocalizations	F^∧^	*t* = 1.173, *p* = 0.253
Proximity person	D	*F*_1_ = 0.405, *p* = 0.531
	Lat	*F*_1_ = 0.546, *p* = 0.468
Proximity to exit	D	*F*_1_ = 1.306, *p* = 0.265

∧ *corrected for overdispersion. Significant differences are highlighted in bold. (L, Likelihood; Lat, Latency; D, Duration; F, Frequency)*.

### Novel object test

#### Object-type

Regardless of species and age, animals interacted more often with the toy-dog than the car (glmm: *z* = 4.752; *p* < 0.001). However, no interaction between species and object-type (glmm: *z* = 0.566; *p* = 0.572) nor age and object-type (glmm: *z* = 0.521; *p* = 0.602) emerged in regard to the likelihood of contacting the objects. Similarly, no interaction between species and object-type (glmm: *z* = 0.291; *p* = 0.771) nor age and object-type emerged in the frequency of interacting with the object (glmm: *z* = 1.530; *p* = 0.126). Since object-type did not cause difference between dogs and wolves and how they behaved at the different ages, all further analyses were conducted without specifying object type.

#### Exploration and interaction with the objects

**Figure 3:**
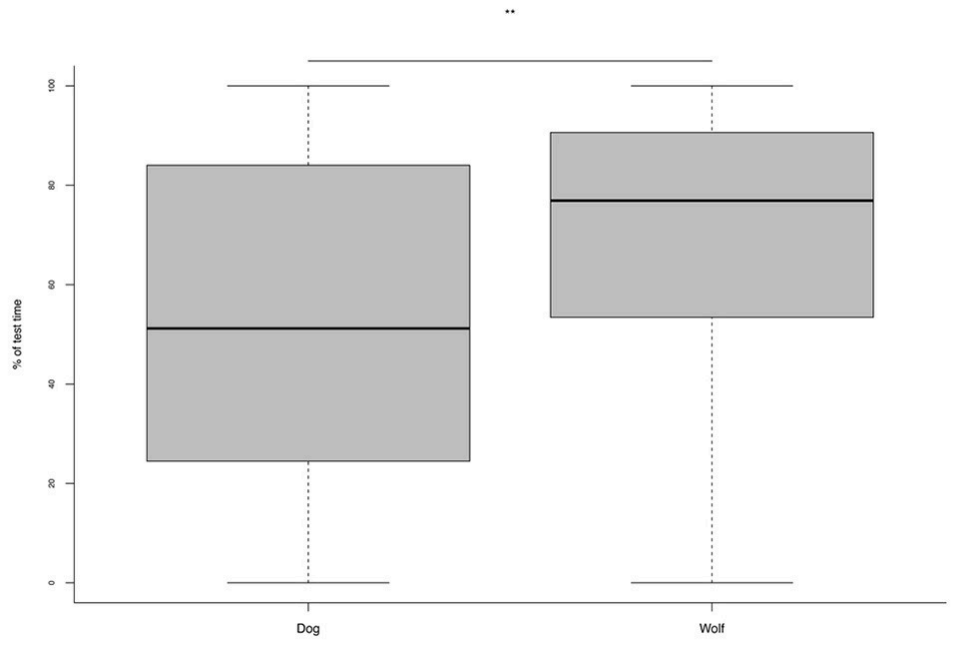
**Percentage of test time spent exploring the environment during the novel object test for wolves and dogs**. ^**^p < 0.001.

Wolves spent significantly more time exploring the environment than dogs (Figure 3) and all animals tended to be more explorative at 8 than at 6 weeks of age (Table 4).

**Table 4:** **Model results regarding explorative behaviors directed at the environment and the object (no of wolves = 17; no of dogs = 18)**.

**Behaviour**	**Measure**	**Factor**	**Model (glmm) statistics**
**Exploration**	D	**Species**	***t*** = **3.66**, ***p*** = **0.0008**
		Age	*t* = 2.25, *p* = 0.025
		Spec^*^Age	*t* = 0.983, *p* = 0.327
Contact object	L	Species	*z* = 1.762, *p* = 0.078
		Age	*z* = 0.674, *p* = 0.500
		Spec^*^Age	*z* = 1.762, *p* = 0.078
Contact object^*^	Lat	Species	$\chi_{\left(1,1\right)}^{2}$ = 0.573, *p* = 0.449
		**Age**	$\chi_{(1,1)}^{2}$ = **10.16**, ***p*** = **0.001**
		Spec^*^Age	$\chi_{\left(1,1\right)}^{2}$ = 2.455, *p* = 0.117
Interact object^*^	F^∧^	Species	***t*** = **1.845**, ***p*** = **0.0651**
		Age	*t* = 1.353, *p* = 0.176
		Spec^*^Age	*t* = 1.044, *p* = 0.296
**Interact object** ^*^	D	**Species**	***t*** = **2.264**, ***p*** = **0.029**
		Age	*t* = 0.572, *p* = 0.569
		Spec^*^Age	*t* = 0.183, *p* = 0.855

∧ corrected for overdispersion;

* *including only animals that performed the behavior. Significant differences are highlighted in bold (L, Likelihood; Lat, Latency; D, Duration; F, Frequency)*.

**Figure 4:**
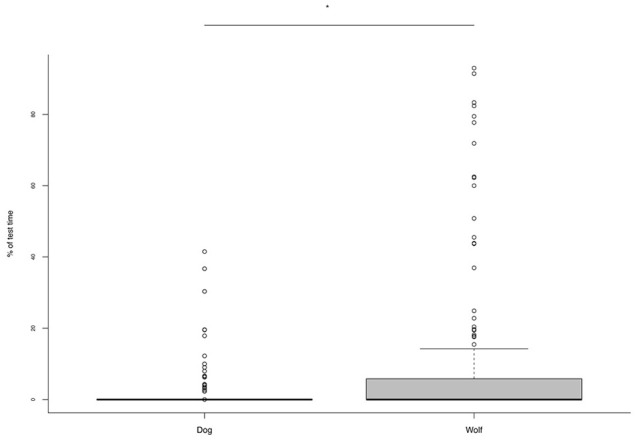
**Percentage of test time spent interacting with the object in dogs and wolves**. ^*^p < 0.01.

As regard to contact with the objects there was a trend for wolves to be more likely to contact the object than dogs (Table 4). Indeed on average at 6 weeks 56% of dogs and 82% of wolves and at 8 weeks 65% of dogs and 88% of wolves contacted the object. Furthermore, wolves interacted with the object more frequently and for longer than dogs (Table 4; Figure 4). For all animals, the latency to contact the object decreased from 6 to 8 weeks of age (Table 4).

#### Stress- and fear- related behaviors, proximity to the exit and vocalizations

An interaction between species and age emerged in the likelihood of exhibiting stress related behaviors. At 6 weeks no wolf-dog difference emerged, with 38% of dogs and 44% of wolves exhibiting stress-related behaviors. At 8 weeks however, wolves were more likely than dogs to show stress-related behaviors, with 67% of wolves and 17% of dogs exhibiting such behaviors. Furthermore, dogs showed a significant decrease in the likelihood of exhibiting stress signals from 6 to 8 weeks of age, whereas wolves showed an opposite pattern, in that they were more likely to exhibit stress signals at 8 than 6 weeks of age (Table 5).

**Table 5:** **Model results regarding stress and fear related behaviours, as well as vocalizations and proximity to the exit (no of wolves = 17; no of dogs = 18)**.

**Behaviour**	**Measure**	**Factor**	**Model (glmm) statistics**
**Stress** **behaviours** -6 weeks -8 weeks Wolves Dogs	L	Spec^*^Age	*z* = **3.846, *p* < 0.001**
		Species	*z* = 0.752, *p* = 0.452
		**Species**	*z* = **4.069, *p* < 0.001**
		**Age**	*z* = **3.724, *p* < 0.001**
		**Age**	*z* = **2.012, *p* = 0.044**
**Stress** **behaviours** -6 weeks -8 weeks Wolves Dogs	F^∧^	Spec^*^Age	*t* = **2.383, *p* = 0.019**
		Species	*t* = 0.96, *p* = 0.343
		**Species**	*t* = **4.502, *p* < 0.001**
		Age	*t* = 1.939, *p* = 0.058
		**Age**	*t* = **2.257, *p* = 0.028**
Fear-related behaviours	L	Species	*z* = 1.079, *p* = 0.28
		Age	*z* = 0.325, *p* = 0.745
		Spec^*^Age	*z* = 0.851, *p* = 0.395
Fear-related behaviours	D	Species	*t* = 1.281, *p* = 0.208
		Age	*t* = 1.131312, *p* = 0.259
		Spec^*^Age	*t* = 1.206, *p* = 0.229
Proximity exit	D	Species	*t* = 1.163, *p* = 0.252
		Age	***t*** **= 1.942**, ***p*** **= 0.053**
		Spec^*^Age	*t* = 1.138, *p* = 0.256
**Vocalizations**	L	**Species**	***z*** **= 1.96**, ***p*** **= 0.049**
		**Age**	***z*** **= 2.370**, ***p*** **= 0.018**
		Spec^*^Age	*z* = 0.022, *p* = 0.982
Vocalizations	F^∧^	Species	*t* = 1.2, *p* = 0.236
		Age	*t* = 1.465, *p* = 0.145
		Spec^*^Age	*t* = 1.127, *p* = 0.262

∧ *corrected for overdispersion. Significant differences are highlighted in bold. (L, Likelihood; Lat, Latency; D, Duration; F, Frequency)*.

**Figure 5:**
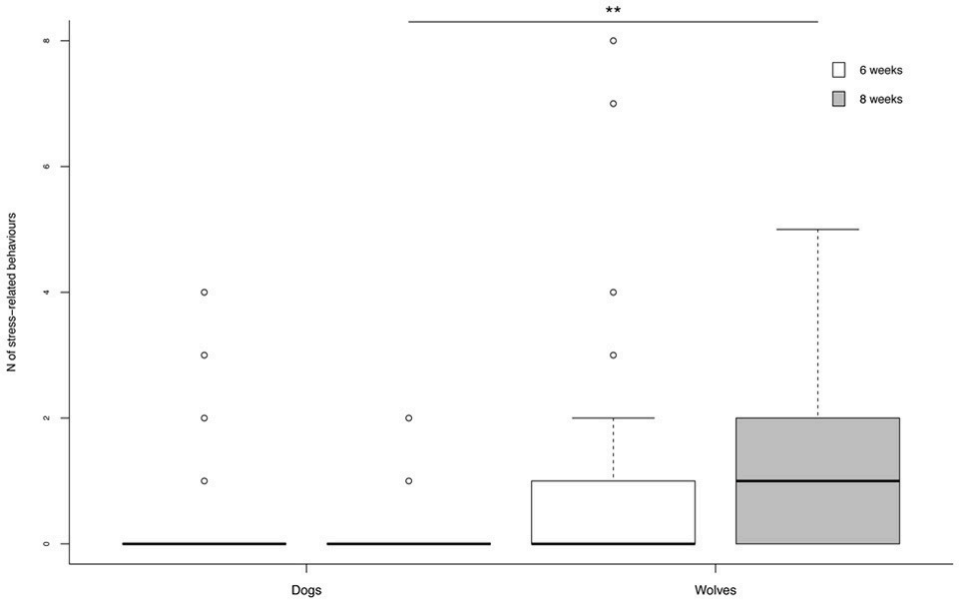
**Frequency of stress-related behaviors exhibited by dogs and wolves during the test at 6 and 8 weeks**. Wolves showed significantly more stress behaviors at 8 than at 6 weeks. ^**^p < 0.001.

A significant age-species interaction also emerged in the frequency of stress behaviors. No species difference was evident at 6 weeks but at 8 weeks wolves showed significantly more stress signals than dogs (Table 5; Figure 5). Taking each species separately, dogs showed a decrease in the frequency of stress signals from 6 to 8 weeks of age, whereas wolves showed a trend in the opposite direction, with more stress signals being exhibited at 8 weeks than 6 weeks of age (Figure 5).

As regard vocalizations, overall animals were less likely to vocalize at 8 than at 6 weeks of age, and wolves were less likely to vocalize than dogs (Table 5). Indeed, at 6 weeks an average of 88% of dogs and 73% of wolves vocalized, whereas at 8 weeks on average 80% of dogs and 67% of wolves showed vocalization behaviors.

No age nor species effects emerged on the exhibition of fear-related behaviors (Table 5). Furthermore, no species differences emerged in the time spent in proximity to the exit. However, overall animals spent less time close to the exit at 8 weeks than 6 weeks (Table 5).

#### Contact experimenter and proximity to people

There was no species effect on the likelihood of contacting the experimenter (Table 6). Indeed on average at 6 weeks 35% of dogs and 58% of wolves and at 8 weeks 40% of dogs and 59% of wolves contacted the experimenter.

**Table 6:** **Model results regarding contact and proximity to people (no of wolves = 17; no of dogs = 18)**.

**Behaviour**	**Measure**	**Factor**	**Model (glmm) statistics**
Contact experimenter	L	Species	*z* = 1.619, *p* = 0.105
		Age	*z* = 0.212, *p* = 0.832
		Spec^*^Age	*z* = 0.236, *p* = 0.814
Contact experimenter^*^	Lat^∧^	Species	$\chi_{\left(1,1\right)}^{2}$ = 0.7, *p* = 0.402
		Age	$\chi_{\left(1,1\right)}^{2}$ = 0.037, *p* = 0.848
		Spec^*^Age	$\chi_{\left(1,1\right)}^{2}$ = 0.047, *p* = 0.827
**Proximity people** -6 weeks -8 weeks Wolves **Dogs**	D	Spec^*^Age	*t* = **2.129, *p* = 0.034**
		Species	*t* = 1.626, *p* = 0.113
		Species	*t* = 0.545, *p* = 0.589
		Age	*t* = 0.643, *p* = 0.521
		**Age**	***t*** = **2.331**, *p* = 0.021

∧ Square root transformed;

* *including only animals that performed the behavior. Significant differences are highlighted in bold. (L, Likelihood; Lat, Latency; D, Duration; F, Frequency)*.

**Figure 6:**
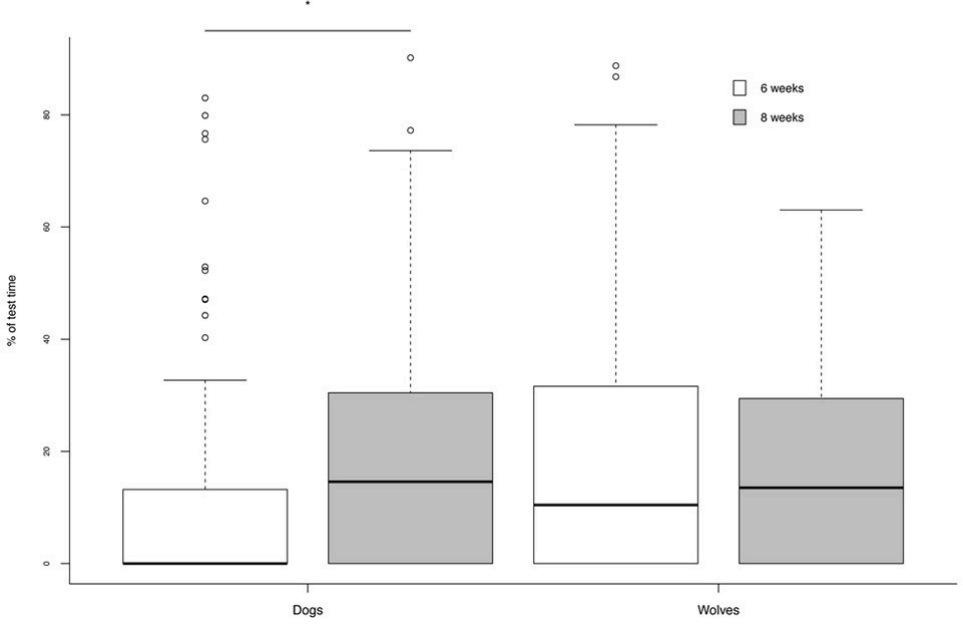
**Percentage of test time spent in proximity to the person**. Whereas dogs showed a significant increase in time spent in proximity to the person from 6 to 8 weeks of age, wolves did not. ^*^p < 0.05.

As regard the time spent in proximity of people (experimenter and camera person summed), an interaction between age and species emerged (Table 6). At 6 weeks wolves had a tendency to spend more time close to the person than dogs, but no such difference was evident at 8 weeks. But whereas dogs increased the time they spent in proximity to the person from 6 to 8 weeks, wolves did not (Table 6; Figure 6).

Overall, considering both tests a consistent pattern of results emerges showing wolves exploring both their environment and novel objects more than dogs (see Table 7 for a summary of the wolf-dog differences emerging).

**Table 7:** **Summary of results pertaining to the wolf-dog comparison in the Novel environment and Novel object tests**.

**Behavior**	**Measure**	**Novel environment (5 weeks)**	**Novel object (6 weeks)**	**Novel object (8 weeks)**
Exploration	D	**Wolves** > **Dogs**	**Wolves** > **Dogs**	**Wolves** > **Dogs**
Self-play	L	Wolves = Dogs	NA	NA
Contact/interaction object	L	NA	**Wolves** > Dogs^*^	**Wolves** > Dogs^*^
	Lat	NA	Wolves = Dogs	Wolves = Dogs
	F	NA	**Wolves** > Dogs^*^	**Wolves** > Dogs^*^
	D	NA	**Wolves** > **Dogs**	**Wolves** > **Dogs**
Contact experimenter	L	NA	Wolves = Dogs	Wolves = Dogs
	Lat	NA	Wolves = Dogs	Wolves = Dogs
Proximity people	D	Wolves = Dogs	Wolves = Dogs	Wolves = Dogs
	Lat	Wolves = Dogs	NA	NA
Stress behaviors	L	NA	Wolves = Dogs	**Wolves** > **Dogs**
	F	Wolves = Dogs	Wolves = Dogs	**Wolves** > **Dogs**
Fear behaviors	L	Wolves = Dogs	Wolves = Dogs	Wolves = Dogs
	D	Wolves = Dogs	Wolves = Dogs	Wolves = Dogs
Proximity exit	D	Wolves = Dogs	Wolves = Dogs	Wolves = Dogs
Vocalizations	L	NA	**Dogs** > **Wolves**	**Dogs** > **Wolves**
	F	Wolves = Dogs	Wolves = Dogs	Wolves = Dogs

* *indicates a trend p < 0.08. L, Likelihood; Lat, Latency; D, Duration; F, Frequency. Species differences are highilighted in bold*.

## Discussion

In line with our predictions wolves explored a new environment more than dogs at the age of 5 weeks and interacted longer with the novel object than dogs both at 6 and 8 weeks. Furthermore, in line with previous results with adults (Moretti et al., 2015), there was a tendency for wolves to be more likely to contact the novel objects than dogs.

A potential reason for these differences is that wolves and dogs have a different developmental trajectory in terms of their motor abilities. Frank and Frank (1982) compared wolves and Malamute dog puppies on a number of problem solving tasks and concluded that wolf pups developed motor skills significantly faster than dogs. For example, wolf pups could climb over their 45 cm pen at 3 weeks of age but Malamute dog pups at 32 days of age were not yet able to climb over their 15 cm barrier. Similarly, at 6 weeks of age wolf pups had much better motor performance than dogs. However, in the present novel object task (both at 6 and 8 weeks of age), there was no difference between wolves and mixed-breed dogs in the latency to contact the object nor the person for those animals that did so. It is therefore possible that middle-sized mixed-breed dogs' motor skills develop differently than that of Malamutes or that the requirements of the current tasks were equally within the animals' motor abilities. It is still possible that especially at 6 weeks, dog pups tired more easily due to their less well-developed motor abilities, thereby influencing the duration of exploration. However, considering also the likelihood of contacting the object tended to differ between wolves and dogs, we suggest that differences in motor abilities are not sufficient to explain the different explorative tendencies of wolf and dog pups. Moreover, the fact that the explorative differences between wolves and dogs we observe in the current study have been found also in older puppies and adults (Topál et al., 2005; Moretti et al., 2015) supports that this behavioral difference is genuine rather than due to differing mobility skills.

As discussed in the introduction, similarly to former studies (e.g., Frank and Frank, 1985, 1987; Miklósi et al., 2003), dogs' reduced exploration of the environment and objects also in the current study may have been a side effect of dogs spending more time in the vicinity/contact with the people present during testing. However, no difference between wolves and dogs emerged either in the likelihood or the latency to contact the people in the room in either test. As for proximity there was a tendency for wolf pups at 6 weeks to spend more time close to the person, although no such difference emerged at 8 weeks. Considering the experimenter was a stranger, it may be that wolves' tendency to spend more time in proximity with her may be in fact an expression of the greater overall explorative tendency observed in wolves compared to dogs (in line with results also from other studies Gácsi et al., 2005; Topál et al., 2005). Although it is possible that the presence of the human affected the explorative behavior of the animals, it is interesting to note that the same pattern of results, with wolves being more explorative than dogs, emerges both in the novel environment test, in which the cameraperson was present but out of sight, and in the novel object test, where the experimenter was quietly standing against the wall. This consistency in the pattern of result would suggest that it is not directly linked to the effect of the human being present.

Another possibility is that dogs explored the object and the environment less because they were more distressed due to separation from the human caregiver and/or their peer group. However, no differences between wolves and dogs emerged in fear-related behaviors and time spent in proximity to the exit. Furthermore, no wolf-dog difference emerged in stress-related behaviors at 5 and 6 weeks and an opposite pattern emerged at 8 weeks, with wolves more likely to exhibit stress-related behaviors and doing so more frequently than dogs. It would therefore seem highly unlikely that dogs' reduced explorative behavior was due to heightened separation anxiety during testing. Our results at week 8, however, are in line with findings of the study conducted with adults in which wolves, although approaching novel objects more readily than dogs, exhibited more fearful behaviors (Moretti et al., 2015). Fear-responses in this former study with adults were specifically related to the object (i.e., walking, running, or jumping away *from the object;* Moretti et al., 2015). Although in the current study, it is unclear whether the differences in frequency of stress behaviors were due to the presence of the object or other elements of the test, e.g., separation from their peers and/or caregiver, the fact that these behaviors became more frequent as the animals got older (and hence less susceptible to separation anxiety) seems to support the former rather than latter explanation.

Overall, based on the current study and in line with the study comparing adult wolves and dogs (Moretti et al., 2015), it appears that domestication has reduced dogs' environmental exploration tendencies and persistence in investigating new objects (as reflected in the shorter duration of interaction time with the novel objects). However, whereas as adults, wolves showed a more intense fear reaction to the novel object compared to dogs (Moretti et al., 2015), this pattern did not emerge as clearly in puppies, although the higher stress-related behaviors in wolves at 8 weeks may be an indication of their neophobic reaction.

The question which remains to be addressed is what factor/s may have changed during domestication, which reduced dogs' persistence in exploring novel objects and also their fear of such objects?

Frank proposed that a consequence of domestication is dogs' reliance on man “as an intermediary between animal and environment” (pp. 272; Frank and Frank, 1985).

In line with this, a number of studies have found that dogs preferentially seek out humans in problem solving tasks, when given such an opportunity (Frank and Frank, 1985; Miklósi et al., 2003; Udell, 2015). Hence one possibility is that during the course of domestication dogs have been selected for a greater dependence/reliance on humans, which has resulted in a reduced motivation for independent explorative behavior.

A second important aspect is that domestication is thought to have affected the timing of an animal's “critical period of development,” i.e., the window of opportunity during which exposure to social and environment stimuli will facilitate acceptance of these as adults (Freedman et al., 1960; Fox and Stelzner, 1966; Lord, 2013 for a review of the dog-wolf literature in particular). This “critical period” is thought to coincide with the onset of mobility (walking and exploration of the environment) and close when animals show avoidance rather than an approach response to novel objects. For example, when comparing foxes selected for tameness to control foxes at 45 days, Trut et al. (2004) found that whereas in the control population there was a decrease in explorative behavior in a new environment and a corresponding increase in both the fearful behaviors and glucocorticoids in the blood, no such changes occurred in the “tame” pups. The “critical period” of the animals selected for tameness, was significantly longer, in that there was no decrease in explorative activity and increase in fear and glucocorticoid levels even at 3 months of age. This extended window of opportunity for exploration with no increase in fear is thought to contribute to the ease with which these animals are socialized to different elements of their environment, including humans.

Wolves' and dogs' critical period has also been reported to differ. A number of authors have reported an increase in avoidance of novelty in wolves at 6 weeks of age (Scott and Marston, 1950; Fentress, 1967; Woolpy and Ginsburg, 1967; Zimen, 1987), whereas in dogs an increase in neophobic behaviors appears to start at around 8 weeks of age, when it is reported that pups will avoid a novel object unless they have been exposed to it for several days (Scott and Marston, 1950; Scott, 1958; Freedman et al., 1960). Our current results are not wholly in agreement with the outlined differential timeline in wolves and dogs. Indeed wolves were more explorative at both 6 and 8 weeks of age, whereas based on the reported information they should have been less explorative at both these times since their critical period should have closed at 6 weeks. Nevertheless, assessment of critical periods can also be based on the expression of stress-related behaviors in general. Considering wolves' showed a higher frequency of stress related behaviors than dogs at 8 weeks, these results are in line with studies suggesting that wild canids' critical period may be shorter than that of dogs. So the increased stress-related behaviors in wolves at 8 weeks is in agreement with the outlined critical period timeline, but the increased explorative behaviors are not. Compared to wolves, dogs showed both less stress behaviors and less exploration of the novel object at 8 weeks. Considering results from the present study with young puppies largely show a similar pattern to those observed with older individuals and adults (Topál et al., 2005; Moretti et al., 2015), it would appear that the wolf-dog differences in explorative behaviors are not limited to a specific developmental phase.

Finally, an often somewhat overlooked aspect which has changed during domestication and has likely affected explorative behaviors, is dogs' ecological niche (Coppinger and Coppinger, 2001, 2016). Indeed, explorative tendencies and neophobia have both been linked to ecological variables in a species environment (Clarke and Lindburg, 1993; Greenberg and Mettke-Hofmann, 2001; Mettke-Hofmann et al., 2002, 2005; Reader and Laland, 2003; Martin and Fitzgerald, 2005). Wolves rely predominantly on group hunting requiring extraordinary persistence considering success rates are between 15 and 50% (Mech et al., 2015). In contrast, free-ranging dogs (i.e., 70–80% of the world's dog population- Lord et al., 2013; Hughes and MacDonald, 2013), live in proximity to human settlements and rely predominantly on solitary scavenging of human waste (Butler and Du Toit, 2002; Butler, 2004; Vanak and Gompper, 2009a,b; Hughes and MacDonald, 2013; Newsome et al., 2014). Human generated food can be considered a more reliable food source compared to live prey, hence in line with past research on other species, the difference in the foraging ecology of wolves and dogs may explain, at least in part the difference in their explorative patterns. While wolves are dependent on greater persistence to obtain their elusive preys (reflected in their greater duration of exploration of novel objects both as pups and adults), dogs as adults at least (Moretti et al., 2015) show less fearful behaviors than wolves when confronted with novel objects, which would be in line with their greater reliance on scavenging in more “humanized” environments.

Whatever the selective pressures affecting wolves' and dogs' differing explorative behaviors (and it is possible that all three aspects combined have played a role in these changes), considering the growing literature highlighting the link between persistence and neophobia on problem solving skills (e.g., Benson-Amram and Holekamp, 2012; Thornton and Samson, 2012), these aspects require further investigation to assess their potential role in domestication and need to be taken into account when comparing the cognitive abilities of wolves and dogs.

## Author contributions

ZV, EK, and FR: Contributed to the conception and design of the study and critical revision of the manuscript; SM: Contributed to the analyses and interpretation of the data as well as the drafting and revising the manuscript. Approval of the final version was given by all authors.

## Funding

SM and FR were supported by funding from the European Research Council under the European Union's Seventh Framework Programme (FP/2007–2013)/ERC Grant Agreement No. [311870]. ZV was supported by the Vienna Science and Technology Fund (WWTF project CS11-026) and by the Austrian Science Fund (FWF project P21244-B17). EK was supported by the Hungarian Academy of Sciences (MTA 01 031, János Bolyai Research Scholarship). The authors further thank many private sponsors including Royal Canin for financial support and the Game Park Ernstbrunn for hosting the Wolf Science Center. The funders had no role in study design, data collection, and analysis, decision to publish, or preparation of the manuscript.

### Conflict of interest statement

The authors declare that the research was conducted in the absence of any commercial or financial relationships that could be construed as a potential conflict of interest.
